# Association between supportive attitude and adoptive practice of control strategy against COVID-19 amosng college students in China: a cross-sectional study

**DOI:** 10.1186/s12889-021-10752-6

**Published:** 2021-04-26

**Authors:** Dong Shen, Dan Liu, Miaochun Cai, Peiliang Chen, Zhenghe Wang, Yujie Zhang, Zhihao Li, Xiru Zhang, Xianbo Wu, Xingfen Yang, Chen Mao

**Affiliations:** Department of Epidemiology, School of Public Health, Southern Medical University, Guangzhou, 510515 China

**Keywords:** Coronavirus disease 19, College students, Prevention and control strategy, Attitude, Adoption

## Abstract

**Background:**

We investigated college students’ attitude and compliance towards a prevention strategy involving use of non-pharmaceutical interventions (NPIs) against coronavirus disease 2019 (COVID-19).

**Methods:**

We conducted a cross-sectional online survey in four universities in Guangdong Province (China) based on purposive sampling. A self-administered questionnaire was given to College students (CSs) to measure the supportive attitude towards an outbreak control strategy and adoption of NPIs in respondents.

**Results:**

A total of 44,446 CSs participated between 31 January and 10 February 2020; 92.7% of respondents supported the outbreak control strategy. The proportion of respondents who avoided public places, wore a facemask, avoid gatherings, and washed hands more frequently than usual was 94.8, 92.8, 91.2 and 86.9%. respectively. A total of 76.5% respondents adopted all four measures. A supportive attitude was associated with NPI adoption. Students who were female, postgraduate, anxious, and not depressed tended to have a higher supportive attitude and higher chance of NPI adoption.

**Conclusions:**

Higher supportiveness towards the disease control strategy for the Chinese public may lead to higher adoption rate of NPIs. Psychosocial factors were related to a supportive attitude and adoption of the NPI. We believe that our findings could aid policymakers to create NPIs to prevent and control emerging infectious diseases such as COVID-19.

**Supplementary Information:**

The online version contains supplementary material available at 10.1186/s12889-021-10752-6.

## Background

Coronavirus disease 2019 (COVID-19) is caused by severe acute respiratory syndrome coronavirus-2 (SARS-CoV-2) infection. COVID-19 was reported first in Wuhan (Hubei Province, China) in December 2019 [1]. The outbreak developed rapidly into a global pandemic. Up to December 2020, ~ 100,000 cases (including imported cases) had been reported in China. However, in some other countries where the first cases were reported in January 2020, such as the USA, Russia, the United Kingdom, and Kazakhstan, the number of cases has been increasing rapidly, reaching 19 million, 3.1 million, 2.4 million, and 200,000 by December 2020, respectively [2]. The difference in the scale of the epidemic in different countries maybe related to the difference in the attitude of the population to the disease and the measures implemented in those countries.

For emerging infectious diseases, due to a lack of efficacious antiviral agents or vaccine, non-pharmaceutical interventions (NPIs) are the most efficacious interventions to prevent and control their spread [3, 4]. During the epidemic of influenza A (H1N1) in 2009, NPIs helped to decrease transmission [5]. Community participation is particularly important for any NPI strategy to be successful [6–9]. Negative attitudes and practices by the public can impede successful campaigns against the spread of infectious diseases [10, 11].

At the early stage of the COVID-19 outbreak, the National Health Commission of China launched a control strategy containing several measures to prevent and control disease spread: free treatment to patients confirmed to have COVID-19; quarantine for people who were likely to have been exposed to SARS-CoV-2 (separation from the rest of the population); restrictions on travelling in and out of Wuhan City; closing down public entertainment facilities; extending the Spring Festival holidays; and delaying the return to school [12]. In addition to the strategy for the public, the Chinese government and its healthcare departments also gave recommendations and guidelines of NPIs for individuals to follow, such as use of a facemask and washing hands frequently. COVID-19 is an emerging infectious disease, so the public had little knowledge about it. NPI messages were propagated vigorously through television, mobile-telephone messages, social media (e.g., WeChat™), the Internet, and newspapers to all community members.

Under these circumstances, the response and implementation by the public for a new NPI strategy are key elements for epidemic control. Additionally, timely feedback of this information is particularly important for health authorities to improve the overall control strategy.

College students (CSs) are important members of the community. They represent the younger, more receptive part of a population. We evaluated CSs’ support and practice towards the NPI strategy against COVID-19 created by the Chinese government. The relationship between their support and practice in the initial period of the COVID-19 outbreak in China was also evaluated. Previous study has shown a gap between knowledge, attitudes, and behaviors toward some health-related events among people. For example, a study conducted among students in Cameroon showed that, although participants had good knowledge and a positive attitude towards infection by the human immunodeficiency virus, they did not undertake preventive practices [13]. Therefore, we intended to observe how consistent the attitude and behavior of CSs were in this public-health emergency.

## Methods

### Ethical approval of the study protocol and consent to participate

The protocol for information collection from participants was approved by the ethics committee of Southern Medical University (Guangzhou, China). This protocol was undertaken in accordance with the ethical standards noted in the 1964 Declaration of Helsinki and its later amendments. Participants were informed of the purpose and overview of the survey and had to provide written consent before completing the questionnaire.

### Study design

From 31 January to 20 February 2020, we provided a cross-sectional online survey for CSs. A purposive sampling method was employed. We sorted universities in Guangdong into four categories according to their main area of specialty: medical, technology, economic, or comprehensive university. Then, we selected one university with ≥20,000 students in each category as our sample. All the students in the universities were selected as participants of this study. The questionnaire used in our study was self-administered. The content and validity of the questionnaire was designed and assessed by six experienced reviewers: three epidemiologists, two specialists in social medicine, and one college counselor (Additional File 1). The questionnaire was sent through online communities at Southern Medical University and was collected via the website of the latter. The validity of the questionnaire was assessed by experts in public health and epidemiology. All questions had to be answered. The survey was anonymous and consent to participate was not required. The report of this study follows the STROBE statement.

### Measures and definitions

The questionnaire consisted of sociodemographic characteristics, Knowledge, Attitude and Practices (KAP) questions, Center for Epidemiologic Studies Depression Scale (CES-D) for depression testing, and Self-Rating Anxiety Scale (SAS) for anxiety testing [14]. Cronbach’s α was used to test the reliability of each part, which was 0.830, 0.823 and 0.856 for KAP, CES-D, and SAS respectively. The cutoff points of these scales were based on studies described previously [15–17]. All participants were required to answer if they had any chance of being exposed to SARS-CoV-2.

In the questionnaire, we defined a supportive attitude towards control of the outbreak by asking “Do you agree with the country’s prevention and disease control policies?”, which include isolation of close contact, large-scale nucleic-acid testing, and promotion of NPIs. Accordingly, the respondents stated “agree” or “disagree”. Adoption of NPI measures included washing hands frequently, using a facemask following recommendations, avoidance of gatherings, and avoidance of public places. Participants were required to answer “complied” or “not complied” to each question. In addition, we defined respondents who adopted all four interventions as “high compliance” and those who did not as “not high compliance”. The outbreak-control strategy and self-protection guidelines were announced on 25 January 2020. Before investigation, we confirmed that participants were aware of the content of the NPI strategy.

We ascertained if participants had a history of potential exposure to SARS-CoV-2 by asking three questions. That is, whether: (i) they travelled to or through Wuhan in the previous 2 weeks; (ii) their family members had been diagnosed with COVID-19 or quarantined; (iii) their family members had travelled to or through Wuhan in the previous 2 weeks. If the answer to all of these questions was negative, the respondents were defined as having “no exposure history”.

### Data analyses

All data collected were cross-checked and imported into Excel™ (Microsoft, Redmond, WA, USA) in simplified Chinese, coded and translated into English, and analyzed using R 3.6.0 (R Foundation for Statistical Computing, Vienna, Austria). The basic characteristics of respondents were first presented as a number (percentage) for categorical variables.

Then, the difference in a supportive attitude to the COVID-19 control strategy and the difference in prevalence of adoption of this strategy in different subgroups was presented. The absolute risk reduction (ARR), number need to treat (NNT) and their 95% confidence intervals (CIs) were calculated. ARR was the difference of event proportion between two groups, and its 95%CI was computed based on Wilson procedure with a correction for continuity. NNT was the reciprocal of the absolute risk difference (1/ARR), and its 95%CI was obtained by taking reciprocals of the values defining the CI for ARR.

The association between a supportive attitude towards the COVID-19 control strategy and its adoption was examined. The significance of differences was assessed by the χ^2^ test for categorical variables and z-ratio for independent proportions. Multivariable logistic regression was carried out to test the association between a supportive attitude towards the NPI strategy and adoption of its measures. Multi-collinearity was ruled out for all covariates based on the collinearity test. Covariates (age, sex, degree course (undergraduate or postgraduate), primary degree (medicine or non-medicine), anxiety score, and depression score were adjusted. Matching of the propensity score based on logistic regression was undertaken to minimize the potential bias in all subgroups, and age, sex, degree course, primary degree, anxiety score, depression score and history of exposure were adjusted accordingly. *P* < 0.05 was considered significant. Missing data were tested by margin plots and considered to be “missing at random”. All missing data were excluded during each analysis.

## Results

### Characteristics of respondents

From 44,451 respondents, 44,446 questionnaires were eligible, the response rate was 54.1% and the median filling time was 664 s (P_25_–P_75_, 523–868). The mean age of all respondents was 21 ± 2.1 (interquartile range, 19–22) years. Overall, 54.5% of respondents were female, 89.4% were undergraduate, and 29.5% were medical students. Among all respondents, 2138 students had a history of exposure to SARS-CoV-2. Among all students who completed the SAS or CESD questions, 0.6% showed signs of anxiety (score ≥ 50), and 33.1% of students showed signs of depression (score ≥ 16) (Table 1).

**Table 1:** Characteristics of college students in the study, *n* = 44,446

Characteristic	Number (%)
**Age, mean ± SD, years**	21 ± 2.4
**Female**	24,231 (54.5)
**Grade**	
Undergraduate	39,723 (89.4)
Postgraduate	4723 (10.6)
**Primary degree**	
Medicine	13,116 (29.5)
Other	31,330 (70.5)
**History of exposure to COVID-19**	
Yes	2138 (4.8)
No	42,308(95.2)
**Anxiety score**	
< 50	44,207 (99.4)
≥ 50	239 (0.6)
**Depression score**	
< 16	29,756 (66.9)
≥ 16	14,690 (33.1)
**Attitude to the strategy to control the epidemic**	
Supportive	30,040 (92.8)
Not supportive	2340 (7.2)
**Using a facemask in accordance with the NPI**	
Complied	41,264 (92.8)
Not complied	3183 (7.2)
**Washing hands frequently**	
Complied	38,613 (86.9)
Not complied	5833 (13.1)
**Avoidance of public places**	
Complied	42,134 (94.8)
Not complied	2312 (5.2)
**Avoidance of gatherings**	
Complied	40,544 (91.2)
Not complied	3902 (8.8)
**Applied all measures**	
Yes	34,005 (76.5)
No	10,041 (23.5)

Note. Data are number (%) unless stated otherwise

The measures adopted most keenly were avoidance of public places (94.8%), followed by use of a facemask (92.8%), avoidance of gatherings (91.2%) and washing hands frequently (86.9%). Of all respondents, 76.5% reported that they would use all four measures for protection against COVID-19 (Table 1).

### Supportive attitude towards the non-pharmaceutical intervention strategy in different subgroups

Female and postgraduate students showed a slightly higher percentage of a supportive attitude towards the NPI strategy against COVID-19 compared with that from male and undergraduate students (ARR of 2.0, and 1.8%, respectively). Respondents with no sign of depression had a more supportive attitude towards strategies than those who were depressed (94.2% vs. 90.5%, ARR = 3.7%). However, respondents who felt anxious were much more approving towards the NPI strategy (95.8% of respondents) than those who did not feel anxious (23.3%). Those who had been exposed to SARS-CoV-2 tended to have less of a supportive attitude than those who had no exposure history (95.8% vs. 91.5%, ARR = 4.3%).

We compared the adjusted number needed to treat (NNT) in subgroups. The smallest value of NNT was 4 and was found in the anxiety group, which suggested that anxiety was the most important factor influencing the supportive attitude of respondents. The second most important factor was exposure history (NNT = 23) (Table 2).

**Table 2:** Supportive attitude towards a non-pharmaceutical intervention strategy to control COVID-19 among respondents by subgroups

Subgroup	Percentage of supportiveness	ARR (95%CI)	P of ARR	NNT (95%CI)
**Sex**				
Male vs. female	91.8% vs. 93.8%	2.0% (1.4–2.6%)	< 0.001	50 (38–71)
Adjusted**†**	91.8% vs. 93.8%	2.0% (1.4–2.6%)	< 0.001	50 (38–71)
**Degree course**				
Undergraduate vs. postgraduate	92.4% vs. 95.2%	2.8% (2.1–3.5%)	< 0.001	35 (29–48)
Adjusted**†**	93.4% vs. 95.2%	1.8% (0.5–3.0%)	0.003	57 (33–200)
**Primary degree**				
Medicine vs. other	93.2% vs. 92.5%	0.6% (0.1–1.2%)	0.029	157 (83–1000)
Adjusted**†**	92.7% vs. 92.5%	0.2% (−0.4 to 0.8%)	0.55	540 (− 250 to 125)
**Depression score**				
< 16 vs. ≥16	93.8% vs. 90.5%	3.3% (2.6–3.9%)	< 0.001	30 (26–38)
Adjusted**†**	94.2% vs. 90.5%	3.7% (3.0–4.4%)	0.002	27 (23–33)
**Anxiety score**				
< 50 vs. ≥50	92.9% vs. 72.5%	20.4% (14.2–26.6%)	< 0.001	5 (4–7)
Adjusted**†**	95.8% vs. 72.5%	23.3% (16.6–30.0%)	< 0.001	4 (3–6)
**Exposure history**				
No vs. Yes	92.9% vs. 91.5%	1.4% (0.1–2.7%)	0.026	73 (37–1000)
Adjusted**†**	95.8% vs. 91.5%	4.3% (2.8–5.8%)	< 0.001	23 (17–36)

Note: Percentage of supportiveness refers to the proportion of respondents who had a supportive attitude to the NPI strategy to control the spread of COVID-19. ARR, absolute risk reduction. NNT, number needed to treat. P (two-tailed) was based on the z-ratio test. †case was adjusted by matching of the propensity score based on age, sex, degree course, primary degree, depression score, anxiety score, or exposure history

### Adoption of non-pharmaceutical intervention measures in different subgroups

The measure adopted by most respondents was avoidance of public places (94.8%), followed by use of a facemask (92.8%), avoidance of gatherings (91.2%) and hand washing frequently (86.9%) (Table 2). Being female, having a high level of education, not suffering from depression, being anxious, and having a history of exposure positively influenced adoption of NPI measures (Table 4). The most important influencing factor was anxiety (ARR = 28%, NNT = 4), followed by depression (9.7% 10), sex (5.9%, 17) and level of education (4.5%, 22); exposure history had only a small influence (2.9%, 34) (Table 3).

**Table 3:** Adoption of the non-pharmaceutical intervention strategy to control COVID-19 among respondents by subgroups

Subgroup	Percentage of adoption	ARR (95%CI)	P of ARR	NNT (95%CI)
**Sex**				
Male vs. female	73.4% vs. 79.1%	5.7% (4.9–6.5%)	< 0.001	18 (15–20)
Adjusted**†**	73.3% vs. 79.2%	5.9% (4.9–6.8%)	< 0.001	17 (15–20)
**Degree course**				
Undergraduate vs. postgraduate	75.3% vs. 81.6%	6.3% (5.0–7.5%)	< 0.001	16 (13–20)
Adjusted**†**	77.1% vs. 81.6%	4.5% (2.4–6.6%)	< 0.001	22 (15–42)
**Primary degree**				
Medicine vs. other	76.3% vs. 76.7%	0.4% (−0.5 to 1.2%)	0.408	250 (−200 to 83)
Adjusted**†**	74.9% vs. 76.7%	1.7% (0.8–2.7%)	< 0.001	59 (37–125)
**Depression score**				
< 16 vs. ≥16	79.7% vs. 70.0%	9.7% (8.8–10.5%)	< 0.001	10 (10–11)
Adjusted**†**	79.7% vs. 70.0%	9.7% (8.7–10.7%)	< 0.001	10 (9–11)
**Anxiety score**				
< 50 vs. ≥50	76.7% vs. 48.5%	28.1% (21.8–34.5%)	< 0.001	4 (3–5)
Adjusted**†**	76.6% vs. 48.5%	28.0% (19.7–36.3%)	< 0.001	4 (3–5)
**Exposure**				
No vs. yes	76.8% vs. 71.3%	5.5% (3.5–7.5%)	< 0.001	18 (29–13)
Adjusted**†**	74.2% vs. 71.3%	2.9% (0.2–5.6%)	< 0.001	34 (500–18)

Note: Percentage of adoption refers to the percentage of respondents who undertook all four measures (hand hygiene, use of a facemask, avoidance of gatherings, and avoidance of public places). ARR, absolute risk reduction. NNT, number needed to treat. P (two-tailed) was based on the z-ratio test. †case was adjusted by matching of the propensity score based on age, sex, degree course, primary degree, depression score, anxiety score, or exposure history

### Associations between a supportive attitude towards the non-pharmaceutical intervention strategy and adoption of its measures

There were significant associations between approval attitude of the NPI strategy and compliance with all of its measures. People who disagreed with the control strategy had a negative association with wearing a facemask (odds ratio, 1.55; 95%CI 1.42 to 1.68), washing hands frequently (1.57; 1.47 to 1.67), avoidance of gatherings (1.26; 1.17 to 1.36), avoidance of public places (1.48; 1.34 to 1.62) and adoption of all measures (1.33; 1.26 to 1.39) (Table 4).

**Table 4:** Association between a supportive attitude and adoption of control measures for COVID-19 among respondents (OR, 95%CI)

Factor	Using a facemask	Washing hands frequently	Avoidance of gatherings	Avoidance of public places	Adoption of all measures
Supportive	1.00 (reference)				
Not supportive	1.55 (1.42–1.68)	1.57 (1.47–1.67)	1.26 (1.17–1.36)	1.48 (1.34–1.62)	1.33 (1.26–1.39)
Age < 21 years	1.00 (reference)				
Age > 21 years	1.14 (1.06–1.23)	1.02 (0.96–1.08)	0.81 (0.75–0.87)	0.92 (0.84–1.01)	0.93 (0.89–0.98)
Male sex	1.00 (reference)				
Female Sex	0.52 (0.49–0.57)	0.74 (0.71–0.79)	0.54 (0.51–0.58)	0.42 (0.38–0.46)	0.67 (0.64–0.70)
Undergraduate	1.00 (reference)				
Postgraduate	0.72 (0.66–0.81)	0.62 (0.57–0.66)	0.84 (0.77–0.92)	0.97 (0.87–1.09)	0.72 (0.68–0.76)
Medicine	1.00 (reference)				
Others	0.59 (0.51–0.69)	0.68 (0.61–0.76)	0.71 (0.61–0.81)	0.63 (0.52–0.76)	0.67 (0.613–0.73)
Depression score < 16	1.00 (reference)				
Depression score > 16	3.09 (2.31–4.12)	2.42 (1.839–3.17)	3.75 (2.832–4.94)	4.75 (3.501–6.38)	2.37 (1.831–3.07)
Anxiety score < 50	1.00(reference)				
Anxiety score < 50	1.89 (1.75–2.03)	1.77 (1.67–1.87)	1.45 (1.35–1.55)	1.39 (1.27–1.51)	1.68 (1.61–1.75)

Note: Odds ratio (95% confidence interval) was adjusted by age, sex, level of education, primary degree, level of depression, and level of anxiety

## Discussion

We found that 92.7% of respondents had a supportive attitude towards the NPI strategy for control of COVID-19. The survey was completed during the winter vacation in China. All CSs were at home for the holidays, and they had the same access to information as the general population. A high prevalence of support indicates that the NPI strategy had been well publicized. About 5.5% of respondents reported that they disagreed with the NPI strategy. Compared with respondents who agreed with the NPI strategy, the respondents who disagreed tended to be male, have a low level of education, and to suffer from depression. Studies have shown consistently that men express a lower level of concern towards health risks [18, 19]. This may be the reason why men had a lower supportive attitude than that of women in our study. Postgraduate students had a slightly higher supportive attitude about the NPI strategy than that of undergraduate students. Studies on Middle East respiratory syndrome (MERS)-related knowledge, preventive behaviors, and risk perception among nursing students during the MERS outbreak showed that senior nursing students and female students had a supportive attitude and practice of measures to control MERS [20–22]. These studies suggest that intelligence has a positive effect on the attitude towards a health strategy.

Importantly, the psychological status of respondents was linked to a supportive attitude of the NPI strategy. Depression was a negative influencing factor in the supportive attitude of the NPI strategy, whereas anxiety had a positive influence regardless of sex or level of education. This result may have been because people suffering from depression frequently lack interest in life [23]. The reasons why anxiety was related to a supportive attitude to the NPI strategy may have been because COVID-19 caused anxiety in these respondents. Few studies have focused on anxiety and attitude towards intervention measures to control and prevent disease. This suggests that people who have moderate anxiety caused by public-health emergencies can enhance their implementation of relevant control strategies.

The measure adopted by most respondents was avoidance of public places (94.8%), followed by use of a facemask (92.8%), avoidance of gatherings (91.2%) and washing hands frequently (86.9%). Compared with the other three measures, the prevalence of adoption of handwashing was relatively low. Implementation of this measure is related to sanitation facilities: in China, there are not enough facilities for outdoor handwashing. The inconvenience and difficulty of maintaining a NPI measure is a potential obstacle to its adoption. During the epidemic of influenza A (H1N1) in 2009, CSs and the general population had a low acceptance of NPI measures because they disrupted workplace and leisure activities [24, 25].

We found that 76.5% of respondents adopted all four NPI measures. Zottarelli and colleagues showed that in the influenza A (H1N1) epidemic in 2009, when evaluating CSs in the USA, 72.1% of the study cohort reported frequent handwashing, yet only 10.7% avoided public gatherings [26]. In another study conducted in a public university in the USA, the proportion of students who took any self-protective measure against influenza A (H1N1) was 64.9% [24]. Those results indicate that more CSs in China implement NPI measures than CSs in the USA.

There was a positive correlation between supportive attitude and adoption behaviors towards the NPI strategy during the COVID-19 epidemic (Table 4). People with a supportive attitude towards the NPI strategy indicated that they had a supportive attitude of protection against and risk of COVID-19, so it is likely that they would be willing to adopt the measures. A study by Wang and colleagues on the factors that determine adoption of preventive behaviors during the influenza A (H7N9) epidemic revealed that a protective attitude positively influenced an individual’s willingness to take recommended actions [27].

Being female, having a high level of education, and being anxious meant that you were likely to adopt the measures of the NPI strategy. Importantly, respondents who reported having anxiety had a 28% higher chance of adopting the measures than those who were not suffering from anxiety. This finding suggested that the anxiety in CSs may have been caused by COVID-19. These results echo those from a study on perceived risk, anxiety, and behavioral responses in the early phase of the influenza A (H1N1) epidemic in the Netherlands [11]. Depression may negatively influence adoption of NPI measures, which may be because depression made people less willing to take action and be more indifferent about safety.

Medical knowledge did not influence adoption of measures. Interestingly, respondents who did not have an exposure history to COVID-19 patients or epidemic areas had less of a supportive attitude and adoptive behaviors to the measures compared with those who had an exposure history (Tables 3 and 4). This finding may have been due to two reasons. First, if respondents had been exposed but did not develop COVID-19, their risk perception will be reduced, which is also known as “optimism bias”. Parry and colleagues showed that, compared with people who had food poisoning due to *Salmonella* species, people who had not experienced food poisoning due to *Salmonella* species perceived their personal risk from food poisoning to be lower [28]. Second, it may be that our respondents had reduced anxiety about COVID-19; Bults and colleagues showed that anxiety decreased over time in the influenza A (H1N1) epidemic in 2009 [11].

Our study had two main limitations, as a cross-sectional study it may be weak in terms of causation, and there may have been an information bias because the study data were time-sensitive and self-reported. However, our study had three main advantages. First, this survey was done shortly after the COVID-19 outbreak, which might indicate how the respondents would actually react. Second, we analyzed the impact on a supportive attitude and adoptive action towards the NPI strategy by the social demographics and psychological status of CSs, which has been studied scarcely previously. Third, this is the first study focusing on a COVID-19-related supportive attitude and adoption towards an NPI strategy. These results can provide: (i) insights for public-health decision-makers; (ii) helpful information on the NPI measures people are willing to adopt (and the factors affecting adoption) during an emerging epidemic.

## Conclusions

Our results suggest that supportiveness towards the disease control strategy for the Chinese public may lead to higher adoption rate of NPIs for individuals, including more challenging measures such as social distancing. It also suggested that policymakers should pay more attention to people suffering from anxiety and depression, who might be less likely to support the policy or prevention measures. Further research is needed to understand differences in responses from other populations, what might affect a supportive attitude, and how these findings will affect action in future outbreaks of infectious diseases.

## Supplementary Information

**Additional file 1.** Questionnaire investigating KAP on COVID-19 and mental health for college students.

## Data Availability

The authors declare that the data supporting the findings of this study will be shared upon reasonable request to the corresponding author.

## Abbreviations

ARR: Absolute risk reduction
CES-D: Center for Epidemiologic Studies Depression Scale
COVID-19: Coronavirus disease 2019
CSs: College students
MERS: East respiratory syndrome
NPIs: Non-pharmaceutical interventions
NNT: Number needed to treat
OR: Odds ratio
SAS: Self-Rating Anxiety Scale
SARS-CoV-2: Severe acute respiratory syndrome coronavirus-2
95%CI: 95% confidence interval

## References

[1] Zhao D, Yao F, Wang L, Zheng L, Gao Y, Ye J, et al. A comparative study on the clinical features of Coronavirus 2019 (COVID-19) pneumonia with other pneumonias. Clin Infect Dis. 2020;71:756–61. 10.1093/cid/ciaa247 PMC7108162 32161968

[2] Coronavirus Disease (COVID-19) Situation Reports. 2021-04-23T08:28:03.000Z. https://www.who.int/emergencies/diseases/novel-coronavirus-2019/situation-reports. Accessed 23 Apr 2021.452Z.

[3] Cowling BJ, Aiello AE. Public health measures to slow community spread of Coronavirus Disease 2019. J Infect Dis. 2020;221:1749–51. 10.1093/infdis/jiaa123 PMC7184488 32193550

[4] Eccleston-Turner M, Phelan A, Katz R (2019). Preparing for the next pandemic - the WHO's global influenza strategy. N Engl J Med.

[5] Fong MW, Gao H, Wong JY, Xiao J, Shiu EYC, Ryu S, Cowling BJ. Nonpharmaceutical Measures for Pandemic Influenza in Nonhealthcare Settings-Social Distancing Measures. Emerg Infect Dis. 2020;26:976–84. 10.3201/eid2605.190995 PMC7181908 32027585

[6] Herbert M, Riyaz BS, Thangaraj S (2012). Community perception regarding rabies prevention and stray dog control in urban slums in India. J Infect Public Health.

[7] Cowling BJ, Ng DM, Ip DK, Liao Q, Lam WW, Wu JT, Lau JT, Griffiths SM, Fielding R (2010). Community psychological and behavioral responses through the first wave of the 2009 influenza a(H1N1) pandemic in Hong Kong. J Infect Dis.

[8] Krogstad DJ, Ruebush TN (1996). Community participation in the control of tropical diseases. Acta Trop.

[9] Bermejo A, Bekui A (1993). Community participation in disease control. Soc Sci Med.

[10] Bults M, Beaujean DJ, Richardus JH, Voeten HA (2015). Perceptions and behavioral responses of the general public during the 2009 influenza a (H1N1) pandemic: a systematic review. Disaster Med Public Health Prep.

[11] Bults M, Beaujean DJ, de Zwart O, Kok G, van Empelen P, van Steenbergen JE, Richardus JH, Voeten HA (2011). Perceived risk, anxiety, and behavioural responses of the general public during the early phase of the influenza a (H1N1) pandemic in the Netherlands: results of three consecutive online surveys. BMC Public Health.

[12] Bureau ODPC (2020). Compilation of public prevention guidelines for pneumonia prevention and control of new coronavirus infection.

[13] Nubed CK, Akoachere J-FTK. Knowledge, attitudes and practices regarding HIV/AIDS among senior secondary school students in Fako Division, South West Region, Cameroon. BMC Public Health. 2016;16:847. 10.1186/s12889-016-3516-9 PMC4994230 27549185

[14] Wang Z, Yang H, Yang Y, Liu D, Li Z, Zhang X, Zhang Y, Shen D, Chen P, Song W (2020). Prevalence of anxiety and depression symptom, and the demands for psychological knowledge and interventions in college students during COVID-19 epidemic: a large cross-sectional study. J AFFECT DISORDERS.

[15] Dunstan DA, Scott N (2020). Norms for Zung's self-rating anxiety scale. BMC PSYCHIATRY.

[16] Jiang L, Wang Y, Zhang Y, Li R, Wu H, Li C, Wu Y, Tao Q (2019). The Reliability and Validity of the Center for Epidemiologic Studies Depression Scale (CES-D) for Chinese University Students. FRONT PSYCHIATRY.

[17] Stahl D, Sum CF, Lum SS, Liow PH, Chan YH, Verma S, Chua HC, Chong SA (2008). Screening for depressive symptoms: validation of the center for epidemiologic studies depression scale (CES-D) in a multiethnic group of patients with diabetes in Singapore. Diabetes Care.

[18] Morioka R (2014). Gender difference in the health risk perception of radiation from Fukushima in Japan: the role of hegemonic masculinity. Soc Sci Med.

[19] Byrnes J, Miller D, Schafer W (1999). Gender differences in risk taking: a meta-analysis. Psychol Bull.

[20] Zhang Y, Xia T, Huang L, Yin M, Sun M, Huang J, Ni Y, Ni J (2019). Factors influencing user engagement of health information disseminated by Chinese provincial Centers for Disease Control and Prevention on WeChat: observational study. JMIR MHEALTH UHEALTH.

[21] Kim JS, Choi JS (2016). Middle East respiratory syndrome-related knowledge, preventive behaviours and risk perception among nursing students during outbreak. J Clin Nurs.

[22] Choi JS, Kim JS (2016). Factors influencing preventive behavior against Middle East respiratory syndrome-coronavirus among nursing students in South Korea. Nurse Educ Today.

[23] Rosenstrom T, Jokela M (2017). Reconsidering the definition of major depression based on collaborative psychiatric epidemiology surveys. J Affect Disord.

[24] Mitchell T, Dee DL, Phares CR, Lipman HB, Gould LH, Kutty P, Desai M, Guh A, Iuliano AD, Silverman P, Siebold J, Armstrong GL, Swerdlow DL, Massoudi MS, Fishbein DB (2011). Non-pharmaceutical interventions during an outbreak of 2009 pandemic influenza a (H1N1) virus infection at a large public university, April-may 2009. Clin Infect Dis.

[25] Stebbins S, Downs JS, Vukotich CJ (2009). Using nonpharmaceutical interventions to prevent influenza transmission in elementary school children: parent and teacher perspectives. J Public Health Manag Pract.

[26] Zottarelli LK, Sunil TS, Flott P, Karbhari S (2012). College student adoption of non-pharmaceutical interventions during the 2009 H1N1 influenza pandemic: a study of two Texas universities in fall 2009. Prev Med.

[27] Wang FP, Wei JP, Shi X (2018). Compliance with recommended protective actions during an H7N9 emergency: a risk perception perspective. DISASTERS.

[28] Parry SM, Miles S, Tridente A, Palmer SR (2004). Differences in perception of risk between people who have and have not experienced Salmonella food poisoning. Risk Anal.

