# Supplementary material for: Association between supportive attitude and adoptive practice of control strategy against COVID-19 amosng college students in China: a cross-sectional study
Source: BMC Public Health. 2021 Apr 26;21:796. doi: 10.1186/s12889-021-10752-6 (PMC8072096; doi:10.1186/s12889-021-10752-6)
Supplement: Supplementary file 1 — Additional file 1. Questionnaire investigating KAP on COVID-19 and mental health for college students. [file 12889_2021_10752_MOESM1_ESM.docx]

**College students COVID-19 KAP and mental health investigation.**

ALL QUESTIONS ARE SINGLE CHOICES UNLESS OTHERWISE STATED.

1 Your gender (single-choice questions)

| Male. |
| --- |
| Female. |

2 Your age_______

3 student Number .________________

4 Your School:

| Basic Medical School. |
| --- |
| Chinese Medicine College. |
| School of Biomedical Engineering. |
| The School of Pharmacy. |
| School of Public Health. |
| Nursing College. |
| School of Inspection and Biotechnology. |
| School of Health Management. |
| The Academy of Marxism. |
| Outside the Chinese College. |
| Faculty of Forensic Medicine. |
| General Education Department. |
| School of Continuing Education. |
| Institute of International Education. |
| First Clinical Medicine School. |
| Second Clinical College of Medicine. |
| Third Clinical College of Medicine. |
| School of Dental Medicine. |
| Medical College of Rehabilitation. |
| Other training units . |

5 Your major:

6 Your grade:

| undergraduate. |
| --- |
| Master's degree. |
| Ph.D. students. |

7 Are you on a research or professional project

| research. |
| --- |
| Professional . |

7 your enrollment year ____________

9 Your ethnic group

| Han Chinese. |
| --- |
| Ethnic minorities: ： |

10 Your place of birth

11 Your winter-break residence.

| City. |
| --- |
| Towns and cities . |
| Rural. |

12 Your winter-break address: . ..

13 Your address in school：_____________.

14 Are anyone with a new coronavirus pneumonia in your relatives (relatives, spouses or relatives) infected with the new coronavirus?

| Someone has been diagnosed. |
| --- |
| There is a suspected person. |
| No one is infected. |
| I don't know. |

15 In two weeks, do you have a history of residence in Hubei, a history of travel, a history of your routes, and a single choice of questions.

| Have a history of residence. |
| --- |
| Have a history of travel. |
| There is a history of pathways. |
| None of the above. |

15.1 Are the family/friends of you come into contact with living or passing through Hubei within two weeks?

| Yes, yes. |
| --- |
| No. |

16 You had planned to return to school at the time:

17 After the school introduced the policy, do you plan to return to school? .

| 1-5 days delay. |
| --- |
| 6-10 days delay. |
| Delayed for more than 10 days. |
| As planned. |
| Such school notification. |

18 Are you satisfied with the deferred back-to-school policy?

| Yes. |
| --- |
| No. |

18.1 Reasons why you are not satisfied with the delayed return policy . . .

| Delayed course progress. |
| --- |
| Delayed progress on the subject. |
| Delayed writing /sending the article progress. / |
| Delayed progress of the experiment (breeding animals, cells, etc.). ) |
| Delayed progress in finding a job. |
| The issue of buying a ticket / refunding a ticket . |
| Other: |

19 Is the tutor satisfied with the school's delayed return policy?

| Yes, yes. |
| --- |
| No. |
| I don't know. |

20 Does the instructor agree with you to postpone your return to school? .

| Yes, |
| --- |
| No. |
| It doesn't matter. |

20.1 Reasons for disagreeing?

| Delays in the progress of the course. |
| --- |
| Delay the on the progress of the project. |
| Delayed writing/posting progress. |
| Delays in the progress of the experiment (breeding animals, cells). ) |
| Other: |

21 After you delay your return to school, there is no contradiction with your tutor?

| There is a great contradiction. |
| --- |
| There are contradictions. |
| There are some contradictions. |
| There is almost no contradiction. |

21.1 Is the reason for your conflict with your mentor?.

| Delayed course progress. |
| --- |
| Delayed progress on the subject. |
| Delayed writing /sending the article progress. / |
| Delayed progress of the experiment (breeding animals, cells, etc.). ） |
| Other: |

22 Do you think there is a prejudice in the society regarding the return of people in Hubei Province?

| Exists. |
| --- |
| There is no existence. |

23 What do you think of the national restrictions on the return of personnel to school / returning units in Hubei Province?.

| This is an act of bias. |
| --- |
| Understand and prevent the outbreak from spreading. |
| Do not understand, delay work and study. |
| It doesn't matter. |

24 As a member of Hubei and Hubei provinces, are you prejudiced?

| Yes. |
| --- |
| No. |

25 At present, some people in the community are biased against Hubei or people in Hubei Province, how do you think of this behavior?

| Fear of viral infection is understandable, but depressed. |
| --- |
| It should not be prejudiced, it is a kind of human apathy. |
| It makes sense to be discriminated against, but I don't blame them. |
| It doesn't matter. |

24 Are you biased against people of Hubei nationality or in Hubei Province.

| Yes. |
| --- |
| No. |

25 At present, some people in the community are biased against Hubei or people in Hubei Province, how do you think of this behavior?

| Understandably, for fear that they would pass the virus on to others. |
| --- |
| Understandably, there is an act of eating wild animals. |
| They should not be discriminated against, it is a complete apathy of humanity. |
| It has nothing to do with me. |

26 Do you think there is a serious social bias against people diagnosed with the new coronavirus/ suspected?

| Critical. |
| --- |
| More severe . |
| It's not serious. |
| Not serious. |

27 The main distance back to school, which of the following transport stakes do you plan to take?

| Aircraft. |
| --- |
| High-speed rail / motor vehicles . |
| Regular trains. |
| Light Rail / Metro . |
| Long-distance bus |
| Buses. |
| Taxi / Express. |
| Private cars. |

28 Return to school in the city, which of the following main transport? .

| Light Rail / Metro . |
| --- |
| Passenger cars . |
| Buses. |
| Taxi / Express / Windmill. |
| Private cars. |

29 Are you worried about going back to school?

| Worried |
| --- |
| Don't worry. |

29.1 What are the main reasons for your concern? (Multiple-choice questions) .

| Worried about being infected by someone else. |
| --- |
| Worried that their body temperature is too high to be isolated. |
| Worried about the suspension of public transport. |
| Worried about traffic jams / crowding affecting travel plans. |
| Worried about a traffic accident. |
| Other: |

29.2 Are you prepared for personal protection?

| Yes, yes. |
| --- |
| No (Please skip to Question 39) |

29.2.1 Specific protective preparations

| Medical surgical masks. |
| --- |
| N95 and above level mask. |
| Alcohol and other disinfection supplies. |
| Other protections: |

29.3 Will you change your travel plans because of the above concerns?

| I will. |
| --- |
| Not. |

30 Have you heard of the Ministry of Education's Notice on the Extension of The Spring Semester 2020 before completing this questionnaire?

| Yes, yes. |
| --- |
| No (please skip to question 42) |

31 How did you learn about the Department of Education's policy? (Multiple-choice questions)

| The official website . . . |
| --- |
| TV. |
| Mobile social software. |
| Newspapers . . . |
| Friends . . . |
| Schools |
| Other channels: |

32 What is your attitude towards the Department of Education's policy on postponing the start of the 2020 spring semester?

| Support. |
| --- |
| It doesn't matter. |
| Objection. |

33 Does the delayed start policy have any effect on your life and study?

| Yes, I do. |
| --- |
| None. |

33.1 What is the specific impact ? (Multiple-choice questions).

| The return trip is subject to change and it is not convenient to book tickets in advance. |
| --- |
| Holidays have no planning awareness and are not efficient in learning. |
| Extended vacation time and more time for self-study. |
| Extended holidays allow you to be fully reunited with your family. |
| Other impacts: |

33.2 What measures would you like the education department and schools to implement during the extended holiday period? (Multiple-choice questions)

| Online courses with open certification credits (e.g. politics courses, some professional electives, etc.) |
| --- |
| Open a psychological support hotline and online counseling. |
| Pass self-study certification credits. |
| Shorten this summer vacation. |
| No measures are required. |
| Other: |

34 A number of provinces and cities across the country have launched a public health emergency Level I response to the outbreak, do you feel fear, anxiety, panic and other psychological response?

| Yes, yes. |
| --- |
| The part is . |
| One point. |
| Not at all. |

34.1 What do you think are the reasons for your mental reactions such as worry, anxiety, panic, etc.? (Multiple-choice questions).

| Because of the diversity of information, there is nothing to do with. |
| --- |
| The unknown, infectious and certain lethal nature of the disease has not yet been developed. |
| Their own psychological quality is poor. |
| Quarantine. |
| Medical resources such as masks are scarce. |
| Food shortages such as vegetables. |
| The other. _________________ |

35 In the face of this outbreak of new coronavirus outbreaks, who would you prefer to express your psychological stress or mood changes?

| Relatives. |
| --- |
| Teacher. |
| Students. |
| Friends. |
| Psychologist. |
| Other. |

36 Do you think it is necessary for a school or teacher to actively focus on students' psychological stress or mood changes?

| It is necessary. |
| --- |
| There is no need. |

37 Do you feel that you need psychological intervention at the moment?

| It is a great need. |
| --- |
| You need . |
| There are some needs. |
| You don't need . |

38 Do you feel you need to know some psychological knowledge and skills to cope with this outbreak?

| You need . |
| --- |
| You don't need . |

38.1 What do you need to know? (Multiple-choice questions) .

| You need to understand how to mitigate the psychological response. |
| --- |
| You need to understand the common symptoms. |
| You need to know how to seek professional psychological help. |
| Other aspects: |

39 Do you think psychological guidance or intervention should be performed according to the scientific psychological assessment tool rating?

| Yes, yes. |
| --- |
| No. |

39.1 What do you think is the current better form of psychological intervention? (Multiple-choice questions)

| TV / Radio. |
| --- |
| Web-based collective courses. |
| The network is one-on-one dredging. |
| Offline lectures. |
| Offline salon . . . |
| The line is one-on-one dredging. |
| Distribution of promotional materials. |
| The other. _________________* |

40 As far as you know, you think that the main susceptible population of the new coronavirus pneumonia is the.

| The general susceptibility of the population. |
| --- |
| Seniors over 65 years of age. |
| Infants and young children . |
| General adults . |

41 Do you think the asymptomatic new coronavirus carrier is contagious.

| Yes. |
| --- |
| No. |
| I don't know. |

42 For the current information, you think that the following is not the clinical manifestation of the new coronavirus pneumonia is the single topic.

| With fever, fatigue, dry cough as the main performance. |
| --- |
| A small number of patients are accompanied by nasal congestion, runny nose, diarrhea and other symptoms. |
| Heavy-duty, critically ill patients can be medium- to low-heat, or even no obvious fever. |
| Most patients have a poor prognosis. |

43 You believe that the means of transmission that have been identified include the "Multiple Topics"

| Spread by respiratory / droplets. |
| --- |
| Contact communication . |
| Spread by water. |
| Spread by arthropods. |

44 What do you think is the severity of the current outbreak ..

| Is serious and may be more than expected. |
| --- |
| It's controllable and will be fine soon. |
| It is not serious. |
| Not sure. |

45 Are you willing to proactively promote scientific knowledge about pneumonia related to the new coronavirus infection to your friends and family?

| Happy and positive. |
| --- |
| A small number of reminders are forwarded without much talk. |
| There is no will. |
| People around me know more than I do. |

46 What do you think of the national prevention and control policy?

| Is correct and appropriate. |
| --- |
| Correct but too strict. |
| The fuss was made. |
| I haven't thought about it. |

47 Do you have the correct use of masks in accordance with the specifications?

| Know the specifications and use them in strict accordance with the standards. |
| --- |
| Understand the specification but do not comply with it. |
| Do not understand the specification. |
| No mask is worn when you go out. |

48 Do you have proper hand hygiene in accordance with the specifications?

| Know the specification and wash your hands in strict accordance with the specification. |
| --- |
| Understand the specification but do not comply with it. |
| Do not understand the specification. |
| Do not wash hands frequently. |

49 Do you have a reduction in family and friends gathering?

| Do not participate as much as possible. |
| --- |
| Some reduction, but will still participate. |
| Not deliberately controlled. |
| Cannot attend (segregated, friends and relatives refuse to party, etc.) for objective reasons. |

50 Do you have a reduction in travel to public places?

| Only if necessary (e.g. medical treatment, procurement of materials, etc.) and not as much as possible. |
| --- |
| To a certain extent, but still travel (play, party, etc.). |
| Travel is not deliberately controlled. |
| Cannot travel (quarantined, etc. for objective reasons. |

51 In the face of the epidemic, how do you practice patriotism with practical actions? (Multiple-choice questions)

| Don't go out and walk around. |
| --- |
| Don't spread rumors. |
| Protect yourself and your family. |
| Actively forward official information. |
| Do not forward unverified information. |
| Donate materials such as masks. |
| Donations . |
| Take the initiative to carry out popular science publicity. |
| Other: |

51.1 The popularity you've done includes:

| A face-to-face mission to those around you. |
| --- |
| Published an online popular science article. |
| Publish newspaper and magazine articles. |
| Make animated videos and other protective promotional materials. |
| Other forms of the _________________ |

| 52 Sort your needs for your school by priority. Body temperatures are taken in dense places (e.g. libraries). |
| --- |
| Disinfect dormitories, teaching halls, libraries, and other dense places, and publish the disinfection of each site. |
| A channel for the purchase of masks or masks is available. |
| Hand washing, disinfection facilities, such as antitoxic disinfectants, are provided in classrooms and canteens. |
| Hand washing instructions, hand sanitizers, blow dryers, disinfectants and other items are provided in the toilet. |
| Restrict the access of off-campus personnel to and from the campus. |
| Conduct psychological screening. |
| Strengthen health literacy. |

53 How do you tend to eat before the outbreak is eliminated after the start of school?

| Pack in the dining hall and bring it back to your dorm or office. |
| --- |
| Eat in the canteen. |
| Point takeaway. |
| Eat in an off-campus restaurant. |
| Other: |

54 How much time have you been plagued by the following questions in the last two weeks?

|  | Not at all. | There were days. | more than half the time. | Almost every day. |
| --- | --- | --- | --- | --- |
| 1. feeling nervous, anxious or anxious. | . | . | . | . |
| 2. Cannot stop or control concerns. | . | . | . | . |
| 3. Too much worry about all kinds of things. | . | . | . | . |
| 3. It's hard to relax. | . | . | . | . |
| 5. Unable to sit still because of uneasiness. | . | . | . | . |
| 6. Become prone to trouble or impatience. | . | . | . | . |
| 7. feeling afraid, it seemed that something terrible would happen. | . | . | . | . |

55 How often have the following manifestations/symptoms appear in your life over the past two weeks?

|  | Not at all. | There were days. | more than half the time. | Almost every day. |
| --- | --- | --- | --- | --- |
| 1. Do things without interest or little fun. | . | . | . | . |
| 2. Feel depressed, depressed, hopeless. | . | . | . | . |
| 3. It's hard to fall asleep, always awake, or sleeping too much. | . | . | . | . |
| I often feel tired or have no energy. | . | . | . | . |
| 5. Have a bad appetite or eat too much. | . | . | . | . |
| 6. Feel bad, or feel like you're failing, and let yourself or your family down. | . | . | . | . |
| 7. It is difficult to concentrate on things, such as reading newspapers or watching TV. | . | . | . | . |
| 8. Movement or speaking slowly enough to attract attention, or restlessness, moving more than usual. | . | . | . | . |
| 9. There is less than the thought of dying, or in some way hurt yourself. | . | . | . | . |

56 Please read carefully and measure the extent to which you have been affected by these issues in the past month. *

|  | Not at all. | A little. | Medium. | Quite a degree. | Extreme. |
| --- | --- | --- | --- | --- | --- |
| 1. A disturbing memory, thought, or image caused by the experience of a stressful event in the past? | . | . | . | . | . |
| 2. The experience of a stressful event in the past has led to repeated and disturbing dreams? | . | . | . | . | . |
| 3. The experience of a stressful event in the past seems to have happened again and felt (as if you were experiencing it again)? | . | . | . | . | . |
| 3. When something reminds you of a stressful event in the past, you're very upset? | . | . | . | . | . |
| 5. When something reminds you of a stressful event in the past, there are physical reactions (such as palpitations, breathing difficulties, sweating)? )? | . | . | . | . | . |
| 6. Avoid thinking about or talking about past experiences of stressful events or feeling sourcing them? | . | . | . | . | . |
| 7. Avoid activities and situations that remind you of the experience of that stressful event? | . | . | . | . | . |
| 8. Can't remember the important content of stressful experiences? | . | . | . | . | . |
| 9. Lost interest in the activities you used to enjoy? | . | . | . | . | . |
| 10. Feeling alienated or disengaged from others? | . | . | . | . | . |
| 11. Feeling emotionally numb or unable to feel loving to people close to you? | . | . | . | . | . |
| 12. Feels like your future will be suddenly interrupted for some reason? | . | . | . | . | . |
| 13. Difficulty falling asleep or waking up? | . | . | . | . | . |
| 14. Irritable or angry outbursts? | . | . | . | . | . |
| 15. Is it hard to concentrate? | . | . | . | . | . |
| 16. Over-alert or alert? | . | . | . | . | . |
| 17. Sensory neurotic or susceptible? | . | . | . | . | . |

The questionnaire is over, thank you for your support and cooperation!

School of Public Health, Southern Medical University.
